# Urethral diverticulum: an unknown pathology

**DOI:** 10.11604/pamj.2022.41.240.34050

**Published:** 2022-03-23

**Authors:** Hamedoun Larbi, Iliass Hassan

**Affiliations:** 1 Service of Urology, Military Hospital of Instruction Mohamed V, Hay Ryad, Rabat, Morocco

**Keywords:** Urethral, diverticulum, magnetic resonance imaging

## Image in medicine

A 25-year-old female patient, pregnant at 30 weeks’ gestation, with no particular medical history, presented to the clinic with urethral pain. The interrogation reveals dysuria evolving for more than 7 months, with urethral pain progressively increasing in intensity and dyspareunia preventing any sexual act for the couple. The clinical examination in the gynecological position found a rounded swelling developed in the anterior wall of the vagina. The vaginal touch allowed the appreciation of the volume and of the consistency. Also, a small pressure on the mass caused cloudy urine to come out, confirming the communication between the pouch and the urethra. Urethrocystoscopy was impossible for our patient. The magnetic resonance imaging (MRI) objectified a urethral diverticulum of the left postero-lateral wall measuring 22*15 mm. Urethral diverticulum occurs in approximately 0.6-6% of women. Clinically, the most common presenting symptoms are urgency, pollakiuria, burning on urination, and dysuria. The most suggestive symptoms are post-void urine loss and dyspareunia. The diagnosis of the urethral diverticulum showed less aggressiveness and 100% reliability thanks to endo-vaginal ultrasound and MRI. The treatment is based on the complete surgical removal of the urethral diverticula for a definitive cure. The therapeutic attitude for our patient is abstention until delivery, then the patient will be proposed to a surgical cure.

**Figure 1:**
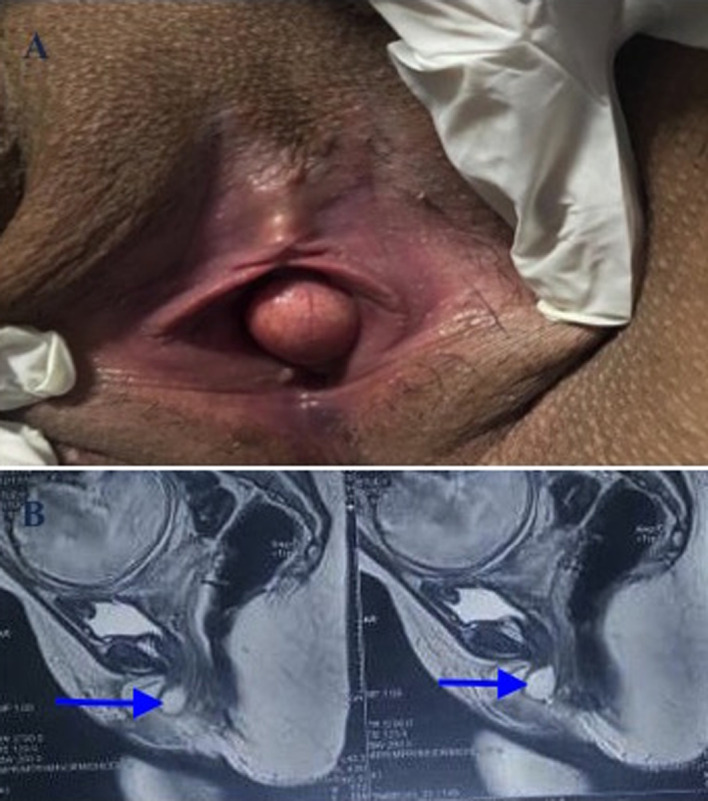
A) clinical appearance of the urethral diverticulum; B) sagittal section of MRI in T2 sequence showing the precise location of the urethral diverticulum

